# Comparison of alternative risk adjustment measures for predictive modeling: high risk patient case finding using Taiwan's National Health Insurance claims

**DOI:** 10.1186/1472-6963-10-343

**Published:** 2010-12-20

**Authors:** Hsien-Yen Chang, Wui-Chiang Lee, Jonathan P Weiner

**Affiliations:** 1 Department of Health Policy & Management, Bloomberg School of Public Health, Johns Hopkins University, 624 N. Broadway, Baltimore, MD 21205, USA; 2 Department of Medical Affairs & Planning, Taipei Veterans General Hospital, and Institute of Hospital Administration & Management, School of Medicine, National Yang-Ming University, 201 Section 2, Shih-Pai Rd, Taipei City 11217, Taiwan

## Abstract

**Background:**

Predictive modeling presents an opportunity to contain the expansion of medical expenditures by focusing on very few people. Evaluation of how risk adjustment models perform in predictive modeling in Taiwan or Asia has been rare. The aims of this study were to evaluate the performance of different risk adjustment models (the ACG risk adjustment system and prior expenditures) in predictive modeling, using Taiwan's National Health Insurance (NHI) claims data, and to compare characteristics of potentially high-expenditure subjects identified through different models.

**Methods:**

A random sample of NHI enrollees continuously enrolled in 2002 and 2003 (n = 164,562) was selected. Health status measures and total expenditures derived from 2002 NHI claims data were used to predict the possibility of becoming 2003 top users. Statistics-based indicators (C-statistics, sensitivity, & Predictive Positive Value) and characteristics of identified top groups by different models (expenditures and prevalence of manageable diseases) were presented.

**Results:**

Both diagnosis-based and prior expenditures models performed much better than the demographic model. Diagnosis-based models were better in identifying top users with manageable diseases; prior expenditures models were better in statistics-based indicators and identifying people with higher average expenditures. Prior expenditures status could correctly identify more actual top users than diagnosis-based or demographic models. The proportions of actual top users that could be identified by diagnosis-based models alone were much lower than that identified by prior expenditures status.

**Conclusions:**

Predicted top users identified by different models have different characteristics and there is little agreement between modes regarding which groups would be potentially top users; therefore, which model to use should depend on the purpose of predictive modeling. Prior expenditures are a more powerful tool than diagnosis-based risk adjusters in terms of correctly identifying more actual high expenditures users. There is still much room left for improvement of diagnosis-based models in predictive modeling.

## Background

Research analyzing the distribution of medical expenditures has consistently shown that a large proportion of medical resources are consumed by a small percentage of the total population[1]. The top 20% of the population with the highest expenditures accounted for about 80% of all healthcare expenditures in the United States[2-4]. This phenomenon of the concentration of healthcare expenditures has been observed continuously since 1970[5]. Consequently, this group of extraordinarily high users of medical resources has inevitably become a target of several types of cost-containment strategies, such as disease management, care management, and utilization review[1,6].

Predictive modeling in health care is generally defined as 'a process of applying existing patient data to prospectively identify persons with high medical needs who are at risk for higher future medical utilization[7].' Predictive modeling is important because early intervention can be delivered to persons identified as possibly having high medical needs. By helping these individuals manage their diseases effectively and providing coordinated medical care, their medical utilization can be reduced and the quality of care they receive can be maintained or improved[8]. In the long run, the expansion of medical expenditures may be controlled within a reasonable range[4,6]. Diagnosis-based health indicators and prior expenditures are the two most common types of risk adjusters for this purpose. It was found that both types of models were very comparable in overall discrimination (by C-statistics), sensitivity, and specificity; however, high-expenditure individuals identified by diagnosis-based models had higher disease burden and somewhat higher healthcare utilization[3,8-10]. Since high-expenditure users identified by diagnosis-based models have more 'manageable' diseases that are targets of disease management programs, it is the preferred model to use.

Taiwan launched a government-run, single-payer National Health Insurance (NHI) programme in May 1995. All Taiwanese nationals are obligated by law to join this programme to ensure adequate risk pooling. Under the jurisdiction of the national government's Department of Health, the NHI is administered by the Bureau of National Health Insurance (BNHI) and six regional branches are in charge of administrating the NHI in each area. The NHI's benefit packages are comprehensive, including inpatient and outpatient services, pharmacy services, Chinese medicine and dental services. Beneficiaries have complete freedom of choice of providers and therapies, and they do not need to go through 'gatekeepers' in order to obtain medical services from specialists. The primary source of funding for the NHI is the payment of premiums shared by the insured, the employers and the government. In terms of reimbursement, the global budget payment system was adopted in order to contain the growth of medical expenditure. Within budget limits, the NHI reimburses contracted providers mostly on a fee-for-service basis, using uniform national fee schedules.

Given the rising concerns to contain the growth of medical expenditures, predictive modeling presents an opportunity to achieve this goal by focusing on very few people. However, previous studies regarding predictive modeling have used regional datasets or focused only on sub-populations, and were conducted in the Western countries. Taiwan is one of very few healthcare systems in the world which has universal coverage and a single national computerized database that includes medical diagnosis information on almost 100% of the population. For this reason the results of this paper have potential policy and methodology implications for most other high or middle income nations.

Few studies related to predictive modeling have been conducted in Taiwan. It has been shown that a government-sponsored disease-management program significantly reduced medical utilization for patients with asthma[11]. In addition, patients in the program had more accurate knowledge of and better self-care skills concerning asthma, and were more likely to adhere to physicians' suggestions[11]. These achievements imply that medical expenditures incurred by this group of patients could potentially be reduced by providing disease and care management, while quality of care could be improved. Utilization review has also been implemented by the Bureau of National Health Insurance (BNHI) in Taiwan, but it was done retrospectively; under such situation, high-expenditure users could only be identified after a large amount of expenditures had occurred, and only a certain proportion of this population would remain high-expenditure users in the following years.

The goal of this study is to evaluate the performance of the Adjusted Clinical Group (ACG) risk adjustment system in predictive modeling using Taiwan's National Health Insurance claims data, and to compare characteristics of potentially high-expenditure subjects identified through different models.

## Methods

### Data sources

The source of the data was a longitudinal dataset prepared by Taiwan's Bureau of National Health Insurance, which is available for researchers interested in observing longitudinal changes of medical utilization. This dataset contained enrollment and claims files of a randomly chosen 1% of Taiwan's population (~200,000 individuals). The enrollment files contained individual subscription information and demographic factors, including sex, date of birth, type of beneficiaries, and location. The claims files contained comprehensive records of inpatient care, ambulatory care, pharmacy store, dental care, and Chinese medicine services. The files also included date of service, ICD-9-CM (International Classification of Diseases) diagnosis codes, claimed medical expenses, and amount of co-payment for each encounter. Twenty-four-month enrollment in both years (2002 and 2003) was required for this analysis, resulting in the final sample size of 164,562 subjects. Individuals' identifiers in this dataset have been encrypted to protect privacy and confidentiality, and this study has been approved by the Johns Hopkins School of Public Health Institutional Review Board.

Annual health expenditures for every NHI enrollee were aggregated from all inpatient, outpatient, and pharmacy store claimed expenses, including claimed reimbursement, medication expenses, and co-payments. Expenses for dental care and Chinese medicine were excluded from this aggregation. Both 2002 and 2003 expenditures were calculated. The unit of money in Taiwan is New Taiwan Dollar (NTD); the exchange rate is about 31 NTD: 1 US dollar as of May 2010. Demographic factors included sex, categorical age (0-17, 18-34, 35-49, 50-64, ≧65), type of beneficiary (insured or dependent), insurance category (based on insured's type of job), residence (three levels with different degrees of population density), and locality (six geographic regions by BNHI's administrative branches: Taipei, Northern, Central, Southern, Kao-Ping, Eastern). Diagnosis-based risk adjustment factors, including ACG, ADG (Aggregated Diagnosis Group) and EDC (Expanded Diagnosis Cluster), were derived from the ACG case-mix system (Version 7.1) using individuals' overall ICD-9-CM codes from both inpatient and outpatient records (diagnosis codes from dental and Chinese medicine services were excluded) in the year 2002.

### The ACG risk adjustment system

ACGs are mutually exclusive health status categories defined by morbidity pattern, age, and sex. The ACG system assigns all ICD-9-CM codes to one of 32 diagnostic clusters (ADGs) based on five clinical dimensions: duration, severity, diagnostic certainty, etiology, and specialty care involvement[12,13]. Each ADG is a grouping of diagnosis codes similar in terms of severity and likelihood of persistence of the health condition treated over a relevant period. ADGs are not mutually exclusive and individuals can have multiple ADGs (up to 32). Individuals are then placed into one of 93 discrete ACG categories according to their assigned ADGs, age, and sex; the result is that individuals within a given ACG have experienced a similar pattern of morbidity and resource consumption. Expanded Diagnosis Clusters (EDCs) are binary indicators to show whether an individual has specific diseases/symptoms. The EDC methodology assigns each ICD code to a single EDC; there are 264 EDCs in total. ICD codes within an EDC share similar clinical characteristics and are expected to induce similar types of diagnostic and therapeutic responses.

### Risk adjustment models

Five risk adjustment models evaluated in this study are listed below based on the comprehensiveness of risk adjusters:

Model 1: Demographics (sex and age groups) only,

Model 2: ACGs with demographics,

Model 3: ADGs plus selected EDCs with demographics,

Model 4: Prior expenditures with demographics, and

Model 5: Prior expenditures, ADGs plus selected EDCs with demographics.

Selected EDCs were derived from the results of stepwise analyses in explaining prospective total expenditures, using a full set of EDCs and a multivariate linear regression model; 19 EDCs were thus chosen (Additional file 1).

### Outcomes and measures of model performance

**Figure 1:**
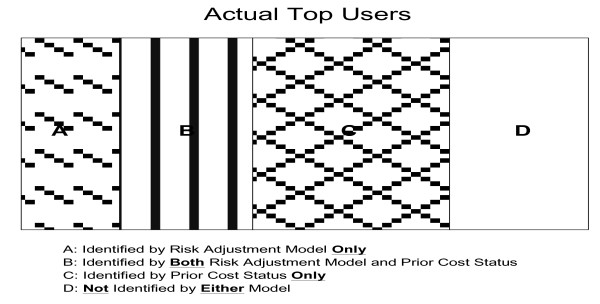
**Classification of actual top users**.

Being a high-expenditure user was a binary variable defined using the following three thresholds: top 0.5%, 1%, and 5% users in 2003. We applied a logistic regression model, given that it is the standard approach to analyze dichotomous outcomes. We conducted all statistical analyses using SAS™ software version 9.1. Performance of five risk adjustment models was evaluated from three aspects: statistical indicators, proportions of true cases identified by models, and characteristics of predicted cases. Statistical indicators included C-statistics, sensitivity, and predictive positive value[1], and the thresholds for calculating statistical indicators were set as the corresponding levels of outcomes. The c-statistic represents the area under the Receiver Operating Characteristic (ROC) curve, and hence provides an overall measure of model performance; in addition, the c-statistic is also independent of other conditions. Actual 2003 top users were assigned to one of four mutually exclusive categories: in 2002 top user group alone, in predicted top user group identified by risk adjustment models alone, in both groups, or in neither group (Figure 1). The real contribution of risk adjustment models comes from those identified by models alone, because these subjects may not be known without applying risk adjustment models (area *a *in Figure 1).

After a group of high-expenditure users was identified by each model, it was also common to examine the characteristics of this population as an alternative method to assess the model performance. Expenditures-based performance indicators included 2003 average total and drug expenditures in the identified top groups, top/bottom total and drug expenditures ratio (2003 average expenditures in top group divided by average expenditures in the rest of the population), and year-2/year-1 total and drug expenditures ratio (2003 average expenditures divided by 2002 average expenditures in top group)[9]. A better risk adjustment model will have higher total and drug expenditures, higher top/bottom expenditures ratio, and higher year-2/year-1 expenditures ratio (subjects with expenditures increasing over time are better targets for intervention). In addition, the proportion of identified high-expenditure subjects with manageable diseases (asthma, COPD, hypertension, depression, or diabetes) is also another important indicator and extensively used[3,10]. Split analysis (a randomly selected 70% of study subjects were used for model development; the rest were set aside for model validation) was performed and measures of model performance were obtained from the validation set to avoid overfitting.

## Results

### Characteristics of the population (Table 1)

**Table 1:** Characteristics of the Taiwanese population for prospective analyses

Inclusion Criteria	2002 & 2003 24 month enrollment
Number of observations	164,562
Male	49.35%
The Insured*	40.93%
Mean age in 2002	34.98
Age Group in 2002	
0 ~ 17	23.84%
18 ~ 34	26.83%
35 ~ 49	25.51%
50 ~ 64	14.19%
≥65	9.62%
**BNHI Branch**	
Taipei	32.52%
Northern	14.81%
Central	19.94%
Southern	14.42%
Kao-Pin	16.03%
Eastern	2.27%
**Residence Level**	
Special Municipality	21.31%
City	15.13%
County	63.56%
**Medical Utilization**	
≥1 outpatient visit	89.71%
≥1 inpatient time	7.17%
≥1 pharmacy expenditures	87.77%
≥1 total expenditures	90.05%
Total expenditures (NTD/yr)	14,741
Medical expenditures (NTD/yr)	10,214
Pharmacy expenditures (NTD/yr)	4,108

*: There are two types of beneficiaries: the insured and the dependent. The dependent obtained its health insurance coverage through the insured.

About half of the study subjects were male and 40% were insured. The mean age in 2002 was 35 years and 10% were elderly. About one-third lived in the areas within the Taipei Branch; only 2% were from the Eastern Branch. About 64% were living in rural county areas. Only 10% had not made any outpatient visit; 7% had at least one inpatient stay. About 90% had non-zero total expenditures and a similar percentage had non-zero drug expenditures. Average annual total expenditures were about 14,700 NTDs, among which medical expenditures (10,200 NTDs) were much high than pharmacy expenditures (4,100 NTDs).

### C-statistics, sensitivity, and positive predictive value (Table 2)

**Table 2:** C-Statistics, sensitivity, and positive predictive value by five risk adjustment models and three outcome thresholds

		Model 1: Demo (sex, ages)	Model 2: ACGs & Demo	Model 3: ADGs, Sel. EDCs & Demo.	Model 4: 2002 expenditures & Demo.	Model 5: ADGs, Sel. EDCs, 2002 expenditures & Demo.
**Outcome: 2003 top 0.5% user (N = 242)**						
C-Statistics		0.773	0.849	0.893	0.904	0.913
	
Top 0.5% predicted group	% identified	0.52%	0.49%	0.48%	0.49%	0.48%
	sensitivity	0.021	0.037	0.343	0.450	0.450
	PPV	0.019	0.037	0.347	0.447	0.456
	
Top 5% predicted group	% identified	5.03%	4.85%	4.89%	4.94%	4.83%
	sensitivity	0.169	0.360	0.665	0.698	0.707
	PPV	0.017	0.036	0.067	0.069	0.072

**Outcome: 2003 top 1% user (N = 492)**						
C-Statistics		0.797	0.860	0.893	0.900	0.907
	
Top 1% predicted group	% identified	0.98%	0.98%	1.01%	1.00%	0.99%
	sensitivity	0.051	0.138	0.313	0.396	0.402
	PPV	0.051	0.140	0.310	0.396	0.403
	
Top 5% predicted group	% identified	4.84%	4.86%	4.88%	4.94%	4.89%
	sensitivity	0.228	0.384	0.598	0.622	0.650
	PPV	0.047	0.079	0.122	0.125	0.133

**Outcome: 2003 top 5% user (N = 2,467)**						
C-Statistics		0.815	0.869	0.884	0.885	0.897
	
Top 5% predicted group	% identified	4.89%	4.89%	4.97%	4.89%	4.93%
	sensitivity	0.258	0.365	0.417	0.467	0.476
	PPV	0.264	0.373	0.419	0.477	0.482

Demo: demographic information (sex and age groups); PPV: positive predictive value

A similar trend was observed across three outcome thresholds (top 0.5%, 1%, and 5% of actual users): more comprehensive models performed better than simpler models in terms of C-statistics. The largest increase was from model 1 to model 2 (~0.06 point), and then from model 2 to model 3 (~0.04 point); the performance of the most comprehensive three models were separated by only 0.01 points. C-statistics in model 1 and 2 increased while those in two prior expenditures models decreased as outcome thresholds were relaxed; those in model 3 remained similar. When the outcome was defined as top 0.5%, 0.1% and 5% of actual 2003 users, c-statistics in model 5 reached 0.913, 0.907, and 0.897, respectively; those in model 1 were 0.773, 0.797 and 0.815, correspondingly.

To calculate sensitivity and PPV, a threshold to define top users was necessary. We used two thresholds in this study for each outcome: the actual proportion of the defined outcomes and the top 5% of identified cases. Results showed that the stricter the threshold, the lower the sensitivity but the higher the PPV. Similarly, sensitivity and PPV went up as the comprehensiveness of the model increased, regardless of outcomes or thresholds. The biggest increase in sensitivity and PPV was from model 2 to model 3, while those in model 4 and 5 were close. When threshold cutoff points were fixed at the top 5%, it showed that as outcome standards relaxed, PPV increased across all models, but sensitivity increased in the demographics-only model and reduced in more comprehensive models.

### Proportion of true cases identified by risk adjustment models and prior top user status (Table 3 & Figure 1)

**Table 3:** Proportion of true cases identified by ACG models and prior expenditures status at three outcome thresholds

		Model 1: Demographics	Model 2: ACGs & Demographics	Model 3: ADGs, Sel., EDCs & Demographics
**Among 2003 top 0.5% user (N = 242)**				
Top 0.5% predicted group	In top 0.5% predicted group only (area A*)	2.07%	2.89%	5.79%
	
	In both groups (area B*)	0.00%	0.83%	28.51%
	
	In 2002 top 0.5% users only (area C*)	47.93%	47.11%	19.42%

**Among 2003 top 1% user (N = 492)**				
Top 1% predicted group	In top 1% predicted group only (area A*)	2.64%	5.08%	6.10%
	
	In both groups (area B*)	2.44%	8.74%	25.20%
	
	In 2002 top 1% users only (area C*)	41.87%	35.57%	19.11%

**Among 2003 top 5% user (N = 2,467)**				
Top 5% predicted group	In top 5% predicted group only (area A*)	10.50%	10.58%	10.82%
	
	In both groups (area B*)	15.28%	25.94%	30.85%
	
	In 2002 top 5% users only (area C*)	32.27%	21.61%	16.70%

*: in Figure 1

We examined three risk adjustment models that were not related to prior expenditures, including model 1 to model 3. Less than half of top users in the current year were also top users in the previous year (47.9%, 44.3%, and 47.6% when the threshold for top users was set as 0.5%, 1%, and 5%, respectively). The proportion of true cases identified by risk adjustment models (Figure 1: area *a *plus area *b*) increased as the comprehensiveness of the risk adjustment model increased, regardless of outcome standards. We also found that the proportion of true cases that could be identified by prior expenditures status was always larger than that proportion by risk adjustment models (Figure 1: area *c *> area *a*), especially in simple ones. In addition, the proportion of true cases identified solely by risk adjustment model (Figure 1: area *a*) was low, and the difference between three models seemed to decrease as the outcome standards relaxed. For example, among 2003 top 1% of actual users, only 5.08% could be identified by the demographics model, 13.82% by model 2, 31.30% by model 3, and the proportion identified by risk adjustment models alone was much lower: 2.64%, 5.08%, and 6.10%, respectively. Among 2003 top 5% of actual users, 25.78% could be identified by the demographic model, 36.52% by model 2 and 41.67% by model 3; the proportion that could be identified by risk adjustment model alone was similar across three models, 10.5%.

### Characteristics of predicted cases by risk adjustment models (Tables 4 &5)

**Table 4:** Expenditure-related characteristics of predicted top user groups identified by five risk adjustment models at three outcome thresholds

		Model 1: Demographics	Model 2: ACGs & Demographics	Model 3: ADGs, Sel. EDCs & Demographics	Model 4: 2002 Expenditures & Demographics	Model 5: ADGs, Sel. EDCs, 2002 Expenditures & Demographics
**Outcome: 2003 top 0.5% user**						
predicted top 0.5% user group	2003 average total expenditures	58,136	108,433	303,755	389,851	391,583
	2003 average drug expenditures	16,706	29,255	41,282	72,792	72,189
	Top/bottom total expenditures ratio	4.1	7.7	23.1	30.7	30.7
	Top/bottom drug expenditures ratio	4.2	7.4	10.6	19.5	19.3
	2003/2002 total expenditures ratio	1.26	0.88	1.03	0.74	0.80
	2003/2002 drug expenditures ratio	1.18	0.88	0.85	0.81	0.84

**Outcome: 2003 top 1% user**						
predicted top 1% user group	2003 average total expenditures	54,520	114,802	220,395	268,398	262,609
	2003 average drug expenditures	16,595	29,367	35,640	59,512	56,430
	Top/bottom total expenditures ratio	3.8	8.5	17.7	22.3	21.7
	Top/bottom drug expenditures ratio	4.2	7.7	9.5	16.9	15.9
	2003/2002 total expenditures ratio	1.20	0.93	1.00	0.76	0.80
	2003/2002 drug expenditures ratio	1.17	0.90	0.89	0.86	0.88

**Outcome: 2003 top 5% user**						
predicted top 5% user group	2003 average total expenditures	51,590	78,295	96,411	110,795	111,235
	2003 average drug expenditures	16,564	23,136	26,830	32,839	32,770
	Top/bottom total expenditures ratio	4.1	6.9	9.4	11.5	11.6
	Top/bottom drug expenditures ratio	4.8	7.5	9.3	12.6	12.6
	2003/2002 total expenditures ratio	1.15	0.99	1.00	0.88	0.91
	2003/2002 drug expenditures ratio	1.09	1.02	1.03	0.98	0.99

**Table 5:** Prevalence of selected diseases among predicted top user groups by five risk adjustment models at three outcome thresholds

	Actual Top Users	Model 1: Demographics	Model 2: ACGs & Demographics	Model 3: ADGs, 19 EDCs & Demographics	Model 4: 2002 Expenditures & Demographics	Model 5: ADGs, 19 EDCs, 2002 Expenditures & Demographics
**2003 Top 0.5% predicted user**						
# of Conditions	0.963	0.988	1.329	1.238	1.160	1.251
Asthma	7.85%	9.27%	12.76%	8.37%	10.25%	8.79%
Hypertension	42.98%	45.56%	55.14%	57.32%	50.82%	54.81%
Depression	6.20%	1.93%	5.35%	3.35%	7.79%	7.95%
Diabetes	21.07%	19.31%	27.57%	33.89%	27.46%	33.05%
COPD	18.18%	22.78%	32.10%	20.92%	19.67%	20.50%

**2003 Top 1% predicted user**						
# of Conditions	0.967	0.971	1.351	1.256	1.270	1.271
Asthma	9.55%	7.61%	14.02%	10.26%	11.99%	11.81%
Hypertension	41.67%	46.91%	55.46%	54.33%	53.05%	52.95%
Depression	6.50%	2.67%	7.63%	7.85%	6.71%	8.76%
Diabetes	20.93%	20.16%	27.84%	30.38%	29.27%	31.57%
COPD	18.09%	19.75%	30.10%	22.74%	26.02%	22.00%

**2003 Top 5% predicted user**						
# of Conditions	1.030	0.981	1.283	1.459	1.288	1.425
Asthma	9.00%	7.50%	11.84%	12.52%	10.39%	12.17%
Hypertension	44.83%	48.38%	57.99%	66.33%	59.73%	64.45%
Depression	6.24%	2.40%	6.13%	8.40%	6.00%	8.30%
Diabetes	26.27%	21.14%	26.78%	34.53%	29.88%	34.32%
COPD	16.62%	18.70%	25.54%	24.13%	22.85%	23.22%

Across three outcome standards, total expenditures, drug expenditures, and top/bottom expenditures ratio generally increased as the comprehensiveness of the risk adjustment model increased. There was a large increase in total expenditures, drug expenditures, and top/bottom expenditures ratios from model 1 to model 2, and from model 2 to model 3; two models with prior expenditures stayed relatively the same. For example, when the outcome was the 2003 top 1% of actual users, the average total expenditures of the predicted top 1% of users increased from 54,520 NTDs in model 1 to 220,395 NTDs in model 3, and then to 262,609 NTDs in model 5. Top/bottom total expenditures ratio increased from 3.8 in model 1 to 18 in model 3, and then to 22 in prior expenditures models (model 4 and 5); top/bottom drug expenditures ratio increased from 4.2 in model 1 to 9.5 in model 3, and then to 16.5 in prior expenditures models. In terms of year-2/year-1 expenditures ratio, model 1 had the highest ratio among all (1.2), model 3 had total expenditures ratio about one, while those of the remaining models were all smaller than one, especially in model 4 and 5 (0.8 ~ 0.9).

As the outcome standard was relaxed from the top 0.5% to the top 5%, there was a general trend for average total and drug expenditures in identified top groups by different models to decrease. Such a decreasing trend became more obvious as the comprehensiveness of risk adjusters increased. For example, the average total expenditures in the demographically-identified top group decreased from 58,000 to 51,590 NTDs, but in the top groups identified by model 5 it decreased from 391,583 to 111,235 NTDs. In addition, the top/bottom expenditures ratio was much higher in total expenditures than in drug expenditures when the outcome was set at the top 0.5% in all but the demographics model (30.7 for total expenditures and 19.3 for drug expenditures in model 5); when the outcome was set at the top 5%, however, the top/bottom drug expenditures ratio was comparable to or higher than the total expenditures ratio (11.6 for total expenditures and 12.6 for drug expenditures in model 5).

In addition to expenditures, we examined the prevalence of five commonly manageable diseases (asthma, hypertension, depression, COPD, and diabetes) among predicted top groups (Table 5). Across three outcome levels, predicted top groups identified by diagnosis-based and prior-expenditures models overall had more manageable diseases than the actual top groups. Those identified by diagnosis-based models had the highest number of manageable conditions compared to models including prior expenditures. On average, the predicted top 0.5% and 1% group by model 2 had 1.33 and 1.35 conditions, respectively; the predicted top 5% group by model 3 had 1.46 conditions; the predicted top groups by the demographic model had the lowest number of conditions across three outcome levels (all less than 1).

When looking at specific conditions, the predicted top groups by diagnosis-based only model generally had higher prevalence of asthma, hypertension, and COPD; those by models including prior expenditures usually had higher prevalence of depression. For example, among the predicted top 1% groups, the prevalence of asthma, hypertension, and COPD were highest in the ACG-identified groups, reaching 14%, 55.5%, and 30%, respectively; the prevalence of depression was highest in the group identified by the model 5, reaching 8.8%. In addition, other than model 1 and 2, the predicted top groups by the remaining three models had higher prevalence of five conditions as the outcome threshold relaxed. For example, among the top groups identified by model 4, the average number of five conditions increased from 1.16, 1.27, to 1.29 while the threshold was set as 0.5%, 1%, and 5%, correspondingly.

Among the predicted top groups across all outcome thresholds and diagnosis-based/prior-expenditures risk adjustment models (demographic model not included), hypertension was the most prevalent condition, with more than 50% having hypertension; diabetes was the second, with about 30%; the third was COPD, ranging from 20% to 30%; ~10% had asthma, and somewhat fewer than 10% had depression.

## Discussion

The results showed that both diagnosis-based and prior expenditures models performed much better than demographic models in predictive modeling, based on virtually all measures evaluated in the study (statistics-based indicators, expenditures indicators, and prevalence of manageable diseases in top groups identified by models). Diagnosis-based models performed better in identifying high-expenditure users with manageable diseases; prior expenditures models were better in statistics-based indicators and identifying people with higher average expenditures. Prior expenditures status could correctly identify more actual high-expenditure users than diagnosis-based or demographics models. The proportions of true high-expenditure users that could be identified by diagnosis-based models alone were much lower than that by prior expenditures status.

In Taiwan, the degree of the concentration of medical expenditures on a small group is comparable to what has been observed in the United States: the top 0.5% consumed somewhat more than 20%, the top 1% consumed about 30%, while the top 5% consumed more than 50% of total medical expenditures. However, the next-year medical expenditures incurred by current high-expenditure users was much higher in Taiwan compared to the United States: in Taiwan, the year 1 top 0.5% group consumed 21.09% in current year and 14.53% in year 2; in the United States, the comparable group consumed about 20% and 7%, respectively. In addition, prior expenditures were also strongly related to current expenditures (Pearson's correlation coefficient between 2002 and 2003 total expenditures: 0.64), and about 50% of high-expenditure users in 2002 remained so in 2003. Therefore, the performance of models including prior expenditures should be better in Taiwan than in the United States. This strong correlation also led to the situation where the top groups identified by models including prior expenditures had higher prior expenditures than those identified by models without prior expenditures. In this study, their year 2 expenditures were higher than year 1, so that it showed a trend of decreasing expenditures over time. Users with expenditures decreasing over time may not be good candidates for interventions because their expenditures are going down already without interventions; they may not even need interventions to bring down their medical expenditures.

On the contrary, diagnosis-based models are better in catching people with 'manageable' conditions and those with increasing expenditures trends. It is critical that subjects predicted to be high-expenditure cases by models are "intervenable" so that their health status and medical utilization can possibly be improved and controlled through managed care or disease management programs. For example, an individual with a serious car accident will have very high medical expenditures in year 1, and will be predicted to be a high-expenditure user in year 2 if prior expenditures are included in the model. It is of little use to identify such a person because nothing much can prevent a car accident from happening (assuming car accidents occur by chance), and his/her medical expenditures will go down naturally in the following year without any intervention.

Part of the reason that the diagnosis-based model is better in catching more people with selected conditions is endogeneity. All five 'manageable' conditions were chronic, and individuals with any of the five conditions were more than likely to have condition-related diagnosis codes on their medical records over a yearly period. Individuals with claims data containing diagnosis codes related to these five conditions were then used as input for diagnosis-based risk adjustment models to identify high-expenditure users. Since patients with any of the five conditions were more likely to consume more resources, they were more likely to be included in identified top groups. And then, the same diagnosis codes were again used to distinguish whether identified subjects had these five conditions.

Without risk adjustment models, the best that health plans can do to identify potentially high-expenditure users is to rely on prior expenditures status. Therefore, the value of risk adjustment models partially lies in the ability to discover what otherwise would not have been found if risk adjustment models were not used. Overall, diagnosis-based models correctly identify a much higher number of high-expenditure users than the demographic model. However, the proportion of actual top users that can be identified solely by diagnosis-based risk adjustment models is only slightly more than what the demographic model can achieve. This is mainly due to the fact that there is a much higher overlap of top predicted groups between diagnosis-based models and prior expenditures status compared to that between the demographic model and prior status (area *b *in Figure 1). Even though diagnosis-based models do not outperform demographic models in identifying more top users with the existence of prior expenditures information, diagnosis-based models are still better choices given the ability to identify subjects with more 'manageable' conditions and higher total expenditures.

It has been shown in the United States that there is little agreement between different models regarding who is identified as top users[3,8-10,14,15]. For example, only 0.19% of total subjects are identified as top users in both prior expenditures and diagnosis-based (DCGs) models when the outcome is set as the top 0.5% (the perfect match would lead to 0.5%)[3]. In Taiwan, the same phenomenon also exists (Table 6). Taking the top 0.5% as an example, the overlap of identified top users between two diagnosis-based models was only 0.07% while that between two models including prior expenditures was also not perfect (only 0.36%). The overlap between the prior expenditures and the comprehensive diagnosis model was 0.19%. Given that there is little overlap and that top groups identified by different models have different characteristics, it is important for policymakers to clarify what the purpose of predictive modeling is before they make decisions on which model to use. If higher total expenditures is preferred, it is crucial to include prior expenditures in the model; if more manageable diseases are preferred, a diagnosis-based model will be more effective.

**Table 6:** Overlap of top predicted groups by different risk adjustment models

	Model 1: Demographics	Model 2: ACGs & Demographics	Model 3: ADGs, Sel. EDCs & Demographics	Model 4: 2002 Expenditures & Demographics	Model 5: ADGs, Sel. EDCs, 2002 Expenditures & Demographics
**2003 Top 0.5% Predicted User**					
Demographics	0.49%*	0.10%	0.03%	0.03%	0.02%
ACGs & Demographics		**0.50%**	0.07%	0.08%	0.06%
ADGs, 19 EDCs & Demographics			**0.50%**	0.19%	0.29%
2002 Expenditures & Demographics				**0.50%**	0.36%
ADGs, 19 EDCs, 2002 Expenditures & Demographics					**0.50%**

**2003 Top 1% Predicted User**					
Demographics	**1.00%**	0.21%	0.11%	0.15%	0.11%
ACGs & Demographics		**1.00%**	0.30%	0.30%	0.32%
ADGs, 19 EDCs & Demographics			**1.00%**	0.40%	0.65%
2002 Expenditures & Demographics				**1.00%**	0.69%
ADGs, 19 EDCs, 2002 Expenditures & Demographics					**1.00%**

**2003 Top 5% Predicted User**					
Demographics	4.96%*	2.43%	2.09%	2.46%	2.01%
ACGs & Demographics		**5.00%**	3.43%	3.02%	3.21%
ADGs, 19 EDCs & Demographics			**5.00%**	3.09%	4.06%
2002 Expenditures & Demographics				**5.00%**	3.73%
ADGs, 19 EDCs, 2002 Expenditures & Demographics					**5.00%**

*: There were several observations with exactly the same predicted value at the threshold.

### Limitations

Predictive modeling presents an opportunity to reduce medical expenditures by identifying a very small number of potential top users regarding whom health plans can take actions. Therefore, identifying potentially top users is only the first step; what will be done after that also plays an important role in determining how much reduction in medical expenditures can possibly be achieved. No matter how perfect the risk adjustment models can be in identifying high-expenditure users, if no effective programs are implemented, the ultimate goal of containing the expansion of medical expenditures will not be realized.

The ACG risk adjustment system was developed using U.S. data; it was not calibrated for how the healthcare system works in Taiwan, so the system may not fit Taiwan's claims data well. However, in prior research it has been shown that the performance of the ACG system in explaining medical utilization in Taiwan was similar to that in other countries[16-18]. In addition, it was also assumed that the claims data obtained from BNHI would be comprehensive enough to capture all important diagnosis codes that may affect the patients' morbidity status. Given comprehensive benefits, easy access to medical care and the low cost of seeking care under NHI, this may not be a big concern in the study.

### Future Research Directions

Many risk adjusters can be used for predictive modeling; however, in this study we included only diagnosis information and prior expenditures. Other than diagnosis information, drug information was also readily available in Taiwan. Therefore, it will be interesting to evaluate how much the performance of risk adjustment models will improve once pharmacy information is also included. It is also better if the model can identify a group of top users with consistently high expenditures over time because it takes time for interventions to show their effectiveness. So, to analyze medical expenditures incurred by predicted top groups from different models--not only in the next year but also several years after--is an important next step to evaluate how risk adjustment models really work.

## Conclusions

Predicted top groups identified by different models have different characteristics and there is little agreement between modes regarding who would be potentially top users. Diagnosis-based models tend to identify people with more 'manageable' diseases; models with prior expenditures are more likely to identify people with higher expenditures. Therefore, which model to use should depend on the purpose of the application. The prior expenditures approach is a more powerful tool than diagnosis-based risk adjusters in terms of correctly identifying actual high-expenditure users. The proportions of actual top users that can be identified by diagnosis-based risk models alone are much lower than those by prior expenditures status alone. There is still much room left for improvement of diagnosis-based models in predictive modeling.

## List of abbreviations

ACG: Adjusted Clinical Group; ADG: Aggregated Diagnosis Group; EDC: Expanded Diagnosis Cluster; BNHI: Bureau of National Health Insurance; ICD: International Classification of Diseases; NTD: New Taiwan Dollar; PM: predictive modeling; PPV: positive predictive value

## Competing interests

While pursuing his PhD at the Johns Hopkins Bloomberg School of Public Health, HYC, the author, worked as a part-time research assistant for Dr. Jonathan Weiner, one of the founders of the ACG system. The author is currently a post-doctoral fellow at Johns Hopkins University, with part of the funding coming from the ACG team. WCL is an ACG researcher and works as a physician manager in Taiwan. JW is one of the developers of the ACG system. The Johns Hopkins University receives royalties for non-academic use of the software based on the ACG methodology.

## Authors' contributions

HYC designed the study, cleaned the data, preformed the statistical analysis, and drafted the manuscript. WCL prepared the claims data and revised the manuscript for publication. JW provided insight into the concept and design of the study and revised the manuscript critically for important intellectual content. All authors read and approved the final manuscript.

## Pre-publication history

The pre-publication history for this paper can be accessed here:

http://www.biomedcentral.com/1472-6963/10/343/prepub

## Supplementary Material

Additional file 1 **Process of generating selected EDCs (Expanded Diagnosis Clusters)**. Describe the process of selecting 19 EDCs for predictive modeling in model 3 & 5. Click here for file
