# Supplementary material for: Comparison of alternative risk adjustment measures for predictive modeling: high risk patient case finding using Taiwan's National Health Insurance claims
Source: BMC Health Serv Res. 2010 Dec 20;10:343. doi: 10.1186/1472-6963-10-343 (PMC3022875; doi:10.1186/1472-6963-10-343)
Supplement: Additional file 1 — Process of generating selected EDCs (Expanded Diagnosis Clusters). Describe the process of selecting 19 EDCs for predictive modeling in model 3 & 5. [file 1472-6963-10-343-S1.DOC]

Additional file 1 – Process of generating selected EDCs (Expanded Diagnosis Clusters)

1. Demographic factors were forced into the model and EDCs entered the model with stepwise selection (significance level for entering in and removing from the model is 0.01).
2. Five types of expenditures (total expenditures, medical expenditures, drug expenditures, inpatient expenditures, outpatient expenditures) were used as dependent variables.
3. For each expenditure outcome, three types of age variables were tested (linear, categorical, and linear splines); therefore, for each expenditure variable, three models were constructed.
4. EDC variables with partial R^2^ greater than or equal to 0.001 were included for further consideration.
5. For each expenditure variable, only EDC variables included in at least two models (out of three different age models) were included for final consideration. Those EDCs on this final list were considered as ‘important’ for this specific expenditure variable.
6. Each EDC variable could be considered ‘important’ at any of five expenditure variables; below was the distribution of number of EDC variables by the count of being considered in ‘important’ category.

| Counts considered ‘important’ in expenditures | 5 | 4 | 3 | 2 | 1 |
| --- | --- | --- | --- | --- | --- |
| Prospective Analyses | | | | | |
| Number of EDC variables | 4 | 2 | 15 | 6 | 9 |
| Number of EDCs with observations <20 | 0 | 0 | 2 | 0 | 2 |

1. EDCs with less than 20 observations were excluded to ensure the stability of the estimates. Four EDCs (NEW04-prematurity, MAL16-Acute Leukemia, INF04-HIV/AIDS, and GUR03-Hypospadias/Other Penile Anomalies) satisfied this exclusion criterion (n=13, 18, 2 and 10, respectively).
2. R^2^ of five models were calculated for prospective analyses.

|  | Model 1 | Model 2 | Model 3 | Model 4 | Model 5 |
| --- | --- | --- | --- | --- | --- |
| Demographics | √ | √ | √ | √ | √ |
| EDCs with counts of importance | 5 | 4, 5 | 3~5 | 2~5 | 1~5 |
| R2 of prospective total expenditures | -- | 17.3% | 18.5% | 18.6% | 18.9% |

1. There was a large drop of R^2^ from Model 3 to Model 2 in prospective analyses. Therefore, EDCs included in Model 3 were chosen.

List of Conditions

| Selected Conditions | Prospective |
| --- | --- |
| Cardiac Arrest, Shock | √ |
| Cardiovascular Disorders, Other | √ |
| Central Nervous System Infections | √ |
| Cerebrovascular Disease | √ |
| Chronic Renal Failure | √ |
| Complications of Mechanical Devices | √ |
| Diabetic Retinopathy | √ |
| Hemophilia, Coagulation Disorder | √ |
| High Impact Malignant Neoplasms | √ |
| Hypertension, w/o Major Complications | √ |
| Ischemic Heart Disease (excl Acute Myocardial Infarction) | √ |
| Malignant Neoplasms, Colorectal | √ |
| Malignant Neoplasms, Liver and Biliary Tract | √ |
| Nephritis, Nephrosis | √ |
| Respiratory Failure | √ |
| Schizophrenia and Affective Psychosis | √ |
| Septicemia | √ |
| Tracheostomy | √ |
| Type 2 Diabetes, w/o Complication | √ |
| Total Number of Conditions | 19 |
